# Evaluation of Bone Marrow Adipose Tissue and Bone Mineralization on Broiler Chickens Affected by Wooden Breast Myopathy

**DOI:** 10.3389/fphys.2019.00674

**Published:** 2019-05-29

**Authors:** Barbara de Almeida Mallmann, Elizabeth M. Martin, Kyung Soo Kim, Norma L. Calderon-Apodaca, Mikayla F. A. Baxter, Juan D. Latorre, Xochitl Hernandez-Velasco, Leopoldo Paasch-Martinez, Casey M. Owens, Sami Dridi, Walter G. Bottje, Elizabeth S. Greene, Guillermo Tellez-Isaias

**Affiliations:** ^1^Department of Poultry Science, University of Arkansas, Fayetteville, AR, United States; ^2^Institute for Nanoscience and Engineering, University of Arkansas, Fayetteville, AR, United States; ^3^Department of Plant Pathology, University of Arkansas, Fayetteville, AR, United States; ^4^Departamento de Medicina y Zootecnia de Aves, Facultad de Medicina Veterinaria y Zootecnia, Universidad Nacional Autónoma de México, Mexico City, Mexico

**Keywords:** bone marrow adipose tissue, broiler chickens, wooden breast, histology, electron microscopy

## Abstract

In humans, alterations in bone metabolism have been associated with myopathies. We postulate the hypothesis that perhaps similar pathologies can also be associated in modern chickens. Hence, this study aimed to assess the fat infiltration in bone marrow and its repercussion on broiler chicken affected by Wooden Breast (WB) myopathy. Ten Cobb 500 live birds with extreme rigidity of the *Pectoralis major* (PM) muscle were selected as WB affected chickens by physical examination of the muscle at 49 days of age, whereas ten chickens healthy with no physical signs of hardness in the breast muscle were considered to be unaffected. Macroscopic lesions in affected chickens included areas of firm and inflamed muscle with pale appearance, hemorrhaging, and viscous exudate on the surface. Bone marrow and sections of the PM muscle were collected and analyzed for light microscopy. Additionally, transmission electron microscopy was conducted in affected or unaffected muscle. Chickens affected with WB showed significant reductions (*P* < 0.05) in femur diameter, calcium, and phosphorous percentage but increased breast weight, compression force and filet thickness when compared with non-affected chickens. Interestingly, bone marrow from WB chicken had subjectively, more abundant infiltration of adipose tissue, when compared with non-affected chickens. Histology of the Pectoralis major of birds with WB showed abundant infiltration of adipose tissue, muscle fibers degeneration with necrosis and infiltration of heterophils and mononuclear cells, connective tissue proliferation, and vasculitis. Ultrastructural changes of WB muscle revealed lack definition of bands in muscle tissue, or any normal ultrastructural anatomy such as myofibrils. The endomysium components were necrotic, and in some areas, the endomysium was notable only as a string of necrotic tissue between degraded myofibrils. The fascia appeared hypertrophied, with large areas of necrosis and myofiber without structural identity with degraded mitochondria adjacent to the disrupted muscle tissue. As far as we know, this is the first study that describes a subjective increase in adipose tissue in the bone marrow of chickens affected with WB when compared with non-affected chickens, and reduced bone mineralization.

## Introduction

The domestic chicken has been an essential animal model and constitutes a remarkable source of high-quality protein for humans (Stern, 2005). Within the last 60 years, genetics of the domestic chicken (*Gallus gallus* domesticus) have been able to create a bird that reaches commercial body weight in 5 weeks (Burt, 2007). Unfortunately, with this astonishing genetic development, other disapproving conditions such as adiposity, leg, metabolic and reproduction pathologies have also increased (Wideman and Hamal, 2011; Abdalla et al., 2018). Recent studies have identified the expression of several genes in the chicken intestine that encodes sugar transporters associated with rapid growth in modern broilers (Miska and Fetterer, 2019). More intriguing are the published studies using ingenuity pathways analysis and histology, comparing the genes of modern broiler chickens suffering from a muscle myopathy called wooden breast (WB), *versus* non-affected chickens, resulted in 1500 genes associated with several disease and physiological disorders (Mutryn et al., 2015; Kuttappan et al., 2017; Marchesi et al., 2018). The WB condition is a myopathy affecting the pectoralis major muscle in fast-growing commercial broiler lines (Velleman and Clark, 2015).

After birth, satellite cells are the most abundant cells in skeletal muscle (Awano et al., 2015). Nevertheless, satellite cells are activated after muscle damage and undergo myogenic differentiation (Carotenuto et al., 2016). The regeneration of the tissue exhibits some resemblances to the development of muscle throughout embryogenesis. Following tissue damage, inflammation and activation of satellite cells, proliferate, differentiate and fuse to form multinucleated myofibers (Karalaki et al., 2009; Lepper et al., 2011). During this process, 5′ AMP-activated protein kinase (AMPK), an enzyme that plays a role in cellular energy homeostasis is also activated (Zhang et al., 2009). However, in some metabolic diseases such as obesity in humans, the reduction of AMPK activity has linked with a significant reduction in muscle regeneration (Kuang et al., 2007). Several investigators have shown that most inflammatory pathways are interconnected in the pathogenesis of diseases affecting the musculoskeletal system in broiler chickens (Mutryn et al., 2015; Wen et al., 2017) and in mammals (Adams, 2002). In chickens, under normal conditions, the skeletal muscle also can repair damage by the activation and differentiation of fiber sub-laminar satellite cells (Halevy et al., 2000). In muscle affected by WB regeneration impairment, due to reduced satellite cells number and/or functional capacity leads to fiber substitution with ectopic tissues including fat and fibrous tissue and the loss of muscle functions (Meloche et al., 2018; Velleman, 2018).

Another critical problem that the current poultry industry is facing is excessive adiposity, which has become an added issue in meat-type chicken production (Wang et al., 2007; Resnyk et al., 2017). While in mammals, lipid tissue regulates energy-balance control throw the secretion of leptin, in chickens, this important adipokine is barely expressed (Matsuzawa et al., 1995). Interestingly, genome array studies have shown that modern chickens suffer from severe obesity (Wang et al., 2006).

Adiposity induces prolonged systemic inflammation, which is associated with several metabolic disorders (Collins et al., 2018). Also, in humans, there is clear evidence indicating the link between chronic inflammation caused by adiposity and myopathies (Zoico et al., 2009; Akhmedov and Berdeaux, 2013; Fu et al., 2016). Nevertheless, there are no reports in modern broiler chickens describing the severe adiposity and its relationship with myopathies.

On the other hand, a recent study has demonstrated the importance of adipose tissue proliferation in the bone marrow in metabolic disorders (Hardouin et al., 2016). Unlike mammals, in birds, fat is added to adipocyte vacuoles without an increase in adipocyte number and do not possess brown fat (King, 1967; Johnston, 1971). A recent study have shown that WB is associated with a significant increase in CO_2_ and a decrease in O_2_. Under this hypoxia conditions or damage defective mitochondria, there is an increase in fatty acids synthesis leading to fatty acid and phospholipid accumulation, responsible of the increased breast fat percentage reported in WB and WS (Ouali et al., 2013; Kozakowska et al., 2015; Livingston et al., 2018). In the bone marrow of fit humans, adipocytes represent seventy percent of total cells. However, aging and other pathological conditions can increase the percentage of adipocytes in the bone marrow (Collins et al., 2018). However, no studies in chickens have evaluated the adipose tissue in the bone marrow and its role in myopathies. Hence, we postulate the hypothesis that the adiposity of modern broiler chickens is associated with an increase fat infiltration in bone marrow, and its repercussion on broiler chicken pectoralis major muscle affected by WB myopathy.

## Materials and Methods

### Animal Source

**FIGURE 1:**
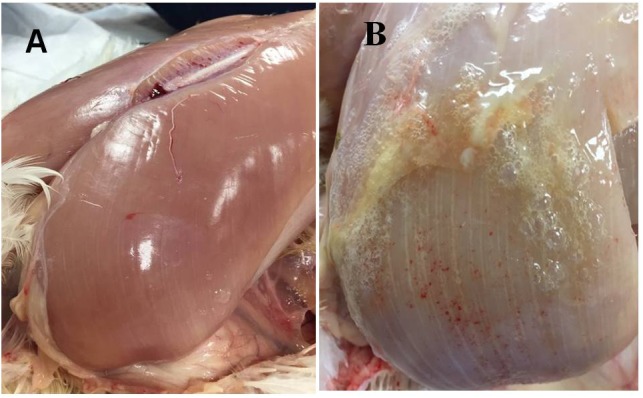
**(A)** *Pectoralis major* non-affected chicken. **(B)** *Pectoralis major* from woody breast chicken. Muscle shows a pale color, hemorrhaging, and a viscous exudate on the surface.

In the present study, male Cobb 500 from Cobb-Vantress (Siloam Springs, AR, United States) were utilized. Ten Cobb 500 live birds with extreme rigidity of the *Pectoralis major* muscle were selected as WB affected chickens by physical examination of the muscle at 49 days of age, whereas ten chickens healthy with no physical signs of hardness in the breast muscle were considered to be unaffected. Macroscopic lesions in affected chickens included areas of firm and inflamed muscle with pale appearance, hemorrhaging, and viscous exudate on the surface (Figure 1). After euthanasia by CO_2_ asphyxiation, breast muscle tissue was harvested from unaffected and affected birds. All animal handling procedures complied with Institutional Animal Care and Use Committee at the University of Arkansas, protocol # 18067.

### Compression Force and Filet Thickness

Compression test parameters were significantly modified (Mudalal et al., 2015) to accommodate for analysis on a whole filet rather than a round cut of a filet. All butterfly filets were split in half with the right filet used for compression analysis and drip loss. Filets were compressed to 20% of the filet height three times on different areas of the cranial region using a 6-mm flat probe on a TA.XT Plus Texture Analyzer (Texture Technologies Corp., Hamilton, MA/Stable Micro Systems, Godalming, Surrey, United Kingdom). No sample cutting was required. The trigger force was set at 5 g, probe height set at 55 mm (higher than the thickest filet sample), pre- and post-probe speeds were both 10 mm/s, and the test speed of the probe was 5 mm/s. The pectoralis major and both tenders were weighed to give an estimate of total meat weight of the breast and the pectoralis major was dissected. Filet thickness was measured at the thickest point with a customized micrometer gauge.

### Bone Parameters

Bone parameters were measured according to the methods as described by previously (Zhang and Coon, 1997). The left femur from the chicken affected or non-affected (*n* = 10) were cleaned of attached tissues. The right femur from the same chickens was used to obtain the bone marrow for histology as described below. The left femurs were dried at 100°C for 24 h and weighed again. The samples were then incinerated in a muffle furnace (Isotemp muffle furnace, Thermo Fisher Scientific, Pittsburgh, PA) at 600°C for 24 h in crucibles. Finally, the content of calcium and phosphorus in the femur was determined using standard methods (AOAC International, 2000) and were reported as a percentage of dry matter.

### Muscle and Bone Marrow Histology

Histological evaluations were performed on five samples of from affected or non-affected chickens immediately after the birds were euthanatized, the skin was removed from the breast region, and a sample of the pectoralis major muscle was excised. Muscle specimens were obtained by dissecting a 0.5-cm-wide area in the anterior portion of the muscle following the muscle fiber orientation for a length of 3 cm. Bone marrow obtained from the right femur and muscle samples were fixed and stored in 10% neutral buffered formalin (vol/vol). Each of these samples was embedded in paraffin, and a 5-μm section of each sample was placed on a glass slide and stained with hematoxylin and eosin for examination under a light microscope.

### Transmission Electron Microscope

Pectoralis major tissue from five affected or non-affected chickens were cut at 2 mm^2^ and fixed with Karnovsky’s fixative in a weak vacuum for 2 h. Samples were rinsed three times with 0.05 M cacodylate buffer pH 7.2, post-fixed for 2 h in 1% osmium tetroxide, with 0.05 M cacodylate buffer. Samples were rinsed in distilled water and stained overnight in 0.5% uranyl acetate at 4.4°C. The tissues were dehydrated in a graded ethanol series then infiltrated with 50:50, Spurr’s medium: 100% ethanol for three changes. Samples were placed in fresh 100% Spurr’s medium, overnight with a weak vacuum. Fresh Spurr’s medium was pipetted into flat embedding molds, and tissues were placed in the molds and aligned. Molds were kept overnight in a weak vacuum. The molds were placed in a 70°C oven overnight. The tissues, in the cured blocks, were trimmed to 1 × 1 mm, and sectioned at 60–90 nm, using a diamond knife with an MT-2B ultra-microtome (Dupont Company, Newtown, CT). Sections were placed on 300 mesh copper grids and stained with 2% aqueous uranyl acetate, followed by lead citrate. Sections were viewed at 100 kV, with a transmission electron microscope (JEM-1011, JEOL, Tokyo, Japan).

### Statistical Analysis

Bone data were subjected to one-way analysis of variance as a completely randomized design using the General Linear Models procedure of SAS (SAS Institute Inc., 2002). Data are expressed as mean ± standard error. Significant differences among the means were determined by using Duncan’s multiple range test at *P* < 0.05.

## Results and Discussion

The growth rates of modern broilers have increased by over 300% (Knowles et al., 2008). However, this intense genetic selection has been accompanied by increased body fat deposition, skeletal disorders, and greater incidence of metabolic diseases and mortality (Wideman and Hamal, 2011). In mammalian models, the molecular and cell and cellular pathways in adipose tissue development is well characterized. Nevertheless, little it is known about broiler chickens (Wang et al., 2017). Genotype, sex, age environmental temperature and nutrition of the broiler chicken are some of the main factors affecting fat deposition (Tůmová and Teimouri, 2010). In animal production, subcutaneous, internal, and intramuscular adipose tissue depots play economically and physiologically essential roles. However, lipid metabolism and adipogenesis in meat animals differ among species. Interestingly, the vertebrates that have a higher capacity for storing and using triglycerides as an energy reserve are found in the class Aves (Blem, 1976). Variation in fatty acid composition among species may be attributed to age, diet and physiological conditions. The amounts of body fat stored as subcutaneous, internal, and intramuscular adipose tissue depots are quantitative traits or complex phenotypes in nature, which are determined by genetic networks or molecular pathways (Hausman et al., 2009; Dodson et al., 2010). In contrast with mammals, lipid accumulation and storage occurs mainly by the addition of lipid to adipocyte vacuoles without an increase in cell number (Blem, 1976). Furthermore, avian fat bodies, unlike those of some mammals, increase in lipid content without similar changes in fat-free dry weight or relative water content of the body (King, 1967), and do not seem to possess brown fat (Johnston, 1971).

The results of the Evaluation of bone parameters and meat quality in 49-days-old broiler chickens non-affected or affected by WB myopathy are summarized in Table 1. Chickens affected with WB showed significant reductions (*P* < 0.05) in femur diameter, calcium, and phosphorous percentage when compared with non-affected chickens (Table 1). Even though body in the present study body weight was not affected between affected or non-affected chickens, chickens affected with WB had a significant increase in breast weight, compression force and filet thickness when compared with non-affected chickens (Table 1).

**Table 1:** Evaluation of bone parameters and meat quality in 49-days-old broiler chickens non-affected or affected by wooden breast myopathy.

	Non-affected	Wooden Breast affected	SEM	*P*-value
**Bone**				
Femur diameter (mm)	12.34^a^	11.02^b^	0.15	0.0032
Calcium (%)	40.48^a^	38.35^b^	0.025	0.0024
Phosphorous (%)	21.15^a^	19.05^b^	1.66	0.0012
**Meat quality**				
Body Weight (kg)	3.52	3.64	0.15	0.5935
Breast Weight (kg)	0.723^b^	0.883^a^	0.025	0.0019
Compression force (N)	6.26^b^	12.89^a^	1.44	0.0115
Filet thickness (mm)	42.86^b^	50.19^a^	1.06	0.0012

*^a,b^Means showing different letters between rows are significantly different. SEM = standard error of the means (n = 10).*

**FIGURE 2:**
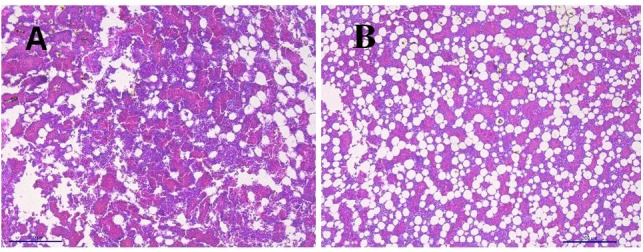
Light microscopy bone marrow. **(A)** Bone marrow non-affected chicken. **(B)** Bone marrow from woody breast chicken with abundant infiltration of adipose tissue.

Figure 2 shows the images of the light microscopy of the bone marrow. Figure 2B shows an image of bone marrow from WB chicken with subjective more abundant infiltration of adipose tissue, when compared with non-affected chicken (Figure 2A).

**FIGURE 3:**
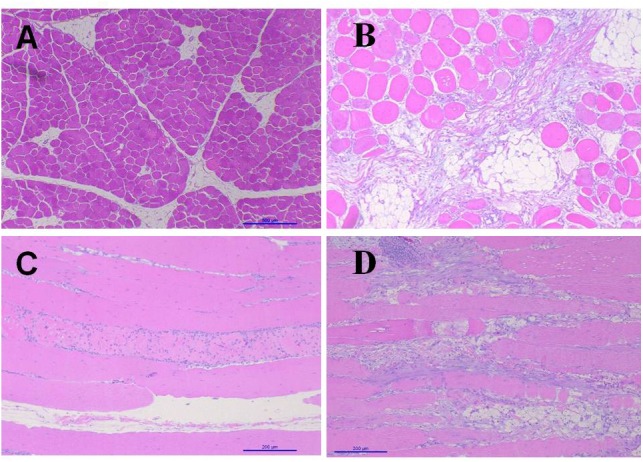
Light microscopy *pectoralis.* **(A)** Transversal cut non-affected chicken. **(B)** Transversal cut woody breast. Abundant infiltration of adipose tissue, muscle fibers degeneration with necrosis and infiltration of heterophiles and mononuclear cells, connective tissue proliferation. **(C)** Longitudinal cut non-affected chicken. **(D)** Longitudinal cut, with interstitial inflammatory infiltrate, vasculitis, muscle fiber degeneration and increase of adipose tissue.

Figure 3 shows the images of the light microscopy of the *Pectoralis major*. In comparison with non-affected chickens, birds with WB showed abundant infiltration of adipose tissue, muscle fibers degeneration with necrosis and infiltration of heterophils and mononuclear cells, connective tissue proliferation, vasculitis, and muscle fiber degeneration (Figure 3B–D). White Striping (WS) is a myopathy categorized by the manifestation of white striations parallel to muscle fibers on breast, thigh, and tender muscles of broilers (Kuttappan et al., 2016). In contrast, WB increases the tougher consistency of the breast. Usually, both these conditions occur in varying degrees on the same filet, and are associated with myodegeneration and necrosis, fibrosis, lipidosis, and regenerative changes (Velleman and Clark, 2015; Velleman et al., 2017). In the present study, non-affected chickens presented, WB eight out of the ten chickens evaluated showed severe lesions of WS when compared with six out of 10 non-affected chickens that presented mild lesions of WS (data not shown).

**FIGURE 4:**
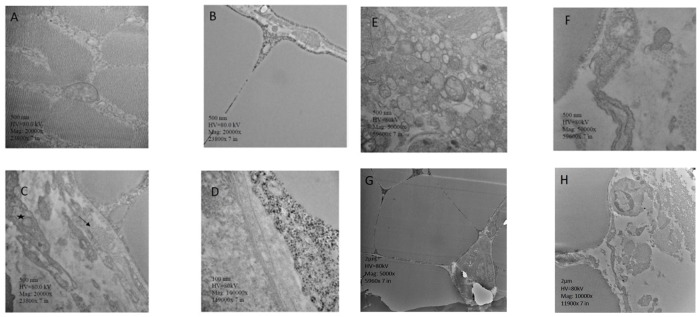
Transmission electron microscopy, *pectoralis major.* **(A)** Muscle fibers with distinct contain myofibrils, in tangential sections. Adjacent endomysium with mitochondria and nucleus apparent are properly arranged. **(B)** Muscle tissue lacks definition of bands, or any characteristic ultrastructural anatomy such as myofibrils. The endomysium components are necrotic, and in some areas, the endomysium is notable only as a string of necrotic tissue between degraded myofibrils. **(C)** Collagen (arrow) noted in the fascia, along with mitochondria. The fascia appears hypertrophied. **(D)** Areas of necrotic myofiber without structural identity. Endomysium contains with degraded mitochondria adjacent to the disrupted muscle tissue. **(E)** Cellular components in the connective tissue are hypertrophied, with noticeable large vesicular material, and areas of separation of components with spongy appearance. **(F)** Cross-section of the collagen within degrading connective tissue, showing degradation of cellular components. Membranes have decomposed. Necrotic tissue is obvious in the adjoining endomysium, and muscle fibers have no noticeable myofibrils. **(G)** Muscle tissue is hypertrophied with lack of noticeable myofibrils. Endomysium tissue components are necrotic, showing only thread-like remains. The adjacent connective tissue appears disrupted. **(H)** Hypertrophied muscle tissue with no noticeable myofibrils. Adjacent collagen in cross-section with disrupted cellular debris. ^∗^Hypertrophied mitochondrion.

The images of the transmission electron microscopy of pectoralis major are presented in Figure 4. Figure 4A shows an image from a non-affected chicken. The lesions observed in the present study are similar to the lesions reported previously (Velleman et al., 1997; Velleman, 2018). The image shows muscle fibers with distinct myofibrils, in tangential sections. Adjacent endomysium with mitochondria and nucleus apparent are appropriately arranged. In contrast, chickens with WB showed lack definition of bands in muscle tissue, or any normal ultrastructural anatomy such as myofibrils. The endomysium components were necrotic, and in some areas, the endomysium was notable only as a string of necrotic tissue between degraded myofibrils. The fascia appeared hypertrophied, with large areas of necrosis and myofibrils without structural identity. Endomysium contains degraded mitochondria adjacent to the disrupted muscle tissue. Furthermore, cellular components in the connective tissue are hypertrophied, with noticeable substantial vesicular material, and areas of separation of components with spongy appearance. Cross-section of the collagen within degrading connective tissue showed degradation of cellular components (Figure 4B–H).

The extraordinary muscle development of modern broilers is going together with increased adipose tissue, predominantly fat adhered to mesentery, along the intestine from the pylorus to the colon (Wang et al., 2007; Leng et al., 2016). Published studies have identified several genes connected with adiposity in chickens, which may help to regulate the excessive accumulation of fat in the gastrointestinal tract (Bourneuf et al., 2006; Bornelöv et al., 2018).

Cells of adipose tissue appear to be highly dynamic since these cells secrete or express many endocrine proteins (Saely et al., 2012; Wensveen et al., 2015). Among them, leptin, a central adipokine that influences growth, metabolism, and behavior the appetite was discovered in the visceral fat of the ob/ob mutant obese mouse (Ingalls et al., 1996). In contrast to mammals, recent studies indicate that in modern chickens, leptin plays a minor role in ruling hungriness, but has a higher endocrine role with the ovary and the testicles (Bornelöv et al., 2018). This biological effect may explain the insatiable appetite of broilers. Modern broilers suffer from severe stress and increase intestinal permeability by depriving the feed for 24 h (Baxter et al., 2017).

In the present study, the increased intramuscular lipid deposits observed at a histologic level in WB lesions are in agreement with several previous reports (Velleman and Clark, 2015; Soglia et al., 2015, 2017). In humans, obesity is associated with an increment of adipose tissue in the muscle, a condition that has been linked with muscle functionally, muscle integrity, and atrophy (Meyer and Ward, 2016). Hence, adipose-based inflammation links adiposity, metabolic disorders and musculoskeletal damage (Zhuo et al., 2012). In mammals, several inflammatory pathways have been linked with myopathies (De Boer et al., 2016; Collins et al., 2018). Under normal conditions, macrophages play a crucial role during early regeneration of damage muscular tissue, which is followed by a series of inflammatory courses, eventually restoring structure and function (Akhmedov and Berdeaux, 2013; Laumonier and Menetrey, 2016). However, the metabolic complications associated with adiposity induce unsuitable recruitment macrophages and hypoxia (Karalaki et al., 2009). Furthermore, adiposity changes the ratio of macrophage type 2 (regenerative) to macrophage type 1 (inflammatory) with increased release of inflammatory cytokines (Wensveen et al., 2015; Thomas and Apovian, 2017; Bijnen et al., 2018). The biggest limitation of the present hypothesis manuscript is that this is a single descriptive study and no mechanistic experiments were conducted to evaluate assays of macrophage function/metabolism. Until today, there are no information of the existence of macrophage type 1 and 2 in chickens.

The extreme hypertrophy in modern broiler chickens has limited space for capillaries in the perimysial connective tissues that result in a poor vascularization (Alnahhas et al., 2016). Hence, a reduction in oxygen and increase in waste products lead to severe oxidative stress and inflammation in the muscle end in myopathies such as WB, spaghetti meat, or white striping (Huang and Ahn, 2018). These changes have been confirmed by RNA-seq analysis, microscopic and biochemical studies. It is now clear that chickens with WB suffer localized hypoxia, increased muscle degradation, reduced glucose utilization, increased intracellular calcium and muscle fiber-type switching (Mutryn et al., 2015; Zambonelli et al., 2016). Furthermore, muscle affected by WB exhibit higher amount of free calcium and sodium as a result of a loss in the intracellular ion homeostasis and an increase of glycolytic activity leading to an increase of pH in affected muscles (Soglia et al., 2018).

On the other hand, mitochondria dysfunction will not only reduce precious energy required for a dynamic tissue such as the muscles but will increase the levels of oxidative stress (Schwarz et al., 2014; Espinosa-Diez et al., 2015). Hence, nutrient overload and adiposity are associated with micronutrient deficiencies, inflammation and lipid peroxidation compromising the integrity of one of the most critical organelles of cells, the cell membrane (Rambold and Pearce, 2018). To further complicate these conditions, obese humans are in a state of chronic hypoxia since adipose tissue has lower capillary vessels, which exacerbates apoptosis, inflammation, and insulin resistance (Engin A, 2017; Engin A.B, 2017).

The alterations in sarcomere and mitochondrial structure, as well as collagen organization reported in WB lesions in the present study, are in agreement with those reported previously (Velleman et al., 1997). Nevertheless, as far as we know, this is the first study that describes a subjective increase in adipose tissue in bone marrow of chickens affected with WB when compared with non-affected chickens, and reduced bone mineralization.

Compared with the other fat depots, in humans, bone marrow adipose tissue plays a crucial role in bone alterations and has been recognized as an essential biomarker of compromised bone integrity (Han et al., 2011; Riondino et al., 2014; Cawthorn and Scheller, 2017). However, analyses of bone marrow adipose tissue development in metabolic diseases and myopathies in chickens are scarce and should be scientifically evaluated to apprehend the role of bone modifications in pathophysiological contexts. It is also important to consider that adipose tissue accumulation in young and adult animals has different implications. Research with meat animals may very well lead to a new understanding of the regulation of lipid metabolism and adipocyte physiology. Because of the propensity to overeat and become obese, the broiler chicken also represents an attractive biomedical model for lipids, energy metabolism, eating disorders and obesity in humans (Wang et al., 2017).

Little is known about macrophages type 1 and types 2 in chickens. Unpublished data from our laboratory suggest that broiler chickens fed with a soy/corn diet exhibit subjectively, infiltration of inflammatory cells that resemble macrophage crowding. If confirmed, this will be a significant breakthrough in chicken immunology. Further studies to evaluate the role of visceral fat, particularly, measurements quantity of fat accumulated in the body of the birds including abdominal fat pad and under skin as well as in the muscle (lipid content of breast muscle), adipokines, and inflammasome, at different ages of broiler chickens are needed to confirm the hypothesis presented in this single and descriptive study.

## Conclusion

Adiposity in modern broilers is a condition that deserves attention. However, there are no studies that have evaluated the relationship between adipose tissue, and the pathways and mechanisms leading to myopathies such as WB. Stress and inflammation can result from a variety of biological, chemical, environmental, or nutritional factors to mention a few. In mammals, adiposity induces chronic systemic inflammatory, a condition linked to several metabolic disorders including cardiovascular and musculoskeletal diseases. Equally important, is the relationship of bone marrow adipose tissue in bone mineralization and hematopoiesis pathologies. The interest in bone marrow adipose tissue is a hot topic because it rises in various pathophysiological diseases. The results of the present hypothesis and theory study suggest that bone marrow adipose tissue and bone mineralization may be linked in the pathogeny caused by WB myopathy in modern broiler chickens.

## Data Availability

All data used in this study are available from the corresponding author upon reasonable request.

## Ethics Statement

All animal handling procedures complied with Institutional Animal Care and Use Committee at the University of Arkansas, protocol # 18067.

## Author Contributions

BdAM, EM, and GT-I designed the experiments and wrote the manuscript. BdAM and EM performed the experiments. MB, KSK, CO, NC-A, WB, and LP-M aided in the analysis and interpretation of data. GT-I, JL, and XH-V contributed to editing the manuscript. CO, SD, WB, EG, and GT-I supervised the project and wrote the manuscript.

## Conflict of Interest Statement

The authors declare that the research was conducted in the absence of any commercial or financial relationships that could be construed as a potential conflict of interest.
